# Kinder und Jugendliche in deutschen Notaufnahmen: Die häufigsten Vorstellungsgründe im Tagesverlauf – aktuelle Erkenntnisse aus dem AKTIN-Notaufnahmeregister

**DOI:** 10.1007/s00063-025-01254-z

**Published:** 2025-03-06

**Authors:** Johanna Bergmann, Alina Balandin, Susanne Drynda, Gunnar Elke, Marcus Klein, Ronny Otto, Domagoj Schunk

**Affiliations:** 1 Interdisziplinäre Notaufnahme und Kindernotaufnahme, Universitätsklinikum Schleswig-Holstein, Campus Kiel, Arnold-Heller-Straße 3, 24105 Kiel, Deutschland; 2 Klinik für Anästhesiologie und operative Intensivmedizin, Universitätsklinikum Schleswig-Holstein, Campus Kiel, Kiel, Deutschland; 3 Universitätsklinik für Unfallchirurgie, Otto-von-Guericke-Universität Magdeburg, Magdeburg, Deutschland

**Keywords:** Pädiatrische Notfälle, Kindernotaufnahme, Kinderverletzungen, Akute Erkrankungen im Kindesalter, Vorstellungszeit, Pediatric emergencies, Pediatric emergency department, Child injuries, Pediatric acute illnesses, Time of presentation

## Abstract

**Hintergrund:**

Die geplante Krankenhaus- und Notfallreform in Deutschland strebt unter anderem eine Umstrukturierung der Notfallversorgung hin zu integrierten Notfallzentren (INZ) und integrierten Notfallzentren für Kinder- und Jugendliche (KINZ) an. Derzeit besteht eine Lücke an aktuellen Daten über die Vorstellungsgründe und die Inanspruchnahme von Notaufnahmen durch Patienten unter 18 Jahren. Die vorliegende Studie bietet eine multizentrische Analyse der häufigsten Vorstellungsgründe von Kindern und Jugendlichen in deutschen Notaufnahmen an.

**Methode:**

In einer retrospektiven, deskriptiven Querschnittsanalyse wurden Daten von 251.570 Notfallpatienten unter 18 Jahren im Zeitraum vom 01.01.2019 bis zum 30.06.2022 aus 22 Notaufnahmen (darunter 3 Kindernotaufnahmen) erfasst. Die Vorstellungsgründe wurden nach dem Canadian Emergency Department Information System—Presenting Complain List (CEDIS-PCL) nach Altersgruppen, Geschlecht und Zuführungsweg ausgewertet.

**Ergebnisse:**

Über 64,1 % der Kinder und Jugendlichen wurden mit einem der 10 häufigsten Vorstellungsgründe vorgestellt. In Kindernotaufnahmen waren überwiegend nichttraumatologische Gründe, wie Atemwegsinfektionen und Bauchschmerzen, dominant, während in zentralen Notaufnahmen (ZNA) vermehrt traumatologische Gründe vorlagen. Die Geschlechterverteilung zeigte bei traumatologischen Gründen eine Mehrheit männlicher Patienten, während bei einigen nichttraumatologischen Vorstellungsgründen, wie Bauch- und Kopfschmerzen, das weibliche Geschlecht überwog. Der überwiegende Teil der Patienten (85,5 %) kam selbstständig in die Notaufnahme; lediglich bei Krampfanfällen dominierte der Rettungsdiensttransport. Im Tagesverlauf sind 67 % der Patienten in der Zeit von 06.00 bis 18.00 Uhr vorstellig, 33 % in den Abend- und Nachtstunden.

**Schlussfolgerung:**

Die Ergebnisse zeigen, dass sich über die Hälfte der Kinder und Jugendlichen mit einem der 10 häufigsten Vorstellungsgründe in den Notaufnahmen vorstellen. Dabei verdeutlichen gerade die nichttraumatologischen Vorstellungen in den ZNA, dass auch in Notaufnahmen für Erwachsene diese Versorgung stattfindet. Das Personal und die Infrastruktur sollten in der Zukunft entsprechend aufgestellt sein, um die Qualität der Kindernotfallversorgung in der Breite effizient zu sichern. Ein wichtiger Ansatzpunkt hierfür ist die Gesundheitsaufklärung und die Optimierung des Zugangs zu ambulanten Versorgungsstrukturen.

**Zusatzmaterial online:**

Zusätzliche Informationen sind in der Online-Version dieses Artikels (10.1007/s00063-025-01254-z) enthalten.

## Abstract (en)

**Background:**

The planned hospital and emergency care reform in Germany aims, among other things, to restructure emergency services towards integrated emergency centers (INZ) and integrated emergency centers for children and adolescents (KINZ). There is a gap in current data on the reasons for presentation and the use of emergency departments by patients under 18 years of age. This study provides a multicenter analysis of the most common reasons for presentation among children and adolescents in German emergency departments.

**Methods:**

In a retrospective, descriptive cross-sectional analysis, data were collected from 251,570 emergency patients under 18 years of age from January 1, 2019, to June 30, 2022, across 22 emergency departments (including three pediatric emergency departments). Reasons for presentation were categorized according to the Canadian Emergency Department Information System—Presenting Complaint List (CEDIS-PCL) and analyzed by age group, gender, and mode of arrival.

**Results:**

Over 64.1% of children and adolescents presented with one of the ten most common reasons. In pediatric emergency departments, nontrauma-related reasons, such as respiratory infections and abdominal pain, were predominant, while trauma-related reasons were more frequent in general emergency departments. The gender distribution showed a majority of male patients for trauma-related reasons, whereas some nontrauma-related reasons, like abdominal and headache complaints, were more common among females. Most patients (85.5%) arrived at the emergency department independently; only for seizures did ambulance transport prevail. During the day, 67% of patients presented between 06:00 and 18:00, with 33% presenting in the evening and nighttime hours.

**Conclusions:**

The results show that more than half of children and adolescents present to emergency departments with one of the ten most common chief complaints. Notably, nontraumatological presentations in emergency departments (EDs) highlight that pediatric care also takes place in facilities primarily serving adults. In the future, staff and infrastructure should be appropriately equipped to efficiently ensure the quality of pediatric emergency care on a broad scale. An important approach in this regard is health education and the optimization of access to outpatient care structures.

## Einleitung

Vor dem Hintergrund der geplanten Krankenhausreform, die einen tiefgreifenden Umbau der Notfallversorgung hin zu integrierten Notfallzentren (INZ) und integrierten Notfallzentren für Kinder- und Jugendmedizin (KINZ) vorsieht [1], ist es von essenzieller Bedeutung, ein detailliertes Verständnis über die gesundheitlichen Gründe für die Inanspruchnahme einer Notaufnahme durch Patienten unter 18 Jahren zu erlangen. Derzeit liegen in Deutschland nur wenige Studien vor, die sich explizit mit der Notfallversorgung von Kindern und Jugendlichen befassen, wobei die bestehenden Untersuchungen überwiegend in Metropolregionen durchgeführt wurden [2]. Um den Umfang des Bedarfs und die Anforderungen an das Gesundheitssystem besser erfassen zu können, bedarf es einer genaueren Betrachtung der aktuellen Situation der Notfallversorgung für Kinder und Jugendliche [2–4]. Dabei müssen Daten zu dieser Zielgruppe erhoben und bewertet werden. Eine pauschale Übertragung der Daten, die bereits über Notfälle im Erwachsenenalter vorliegen, ist nicht möglich [2].

Ziel dieser multizentrischen Studie war es, auf der Basis von Behandlungsdaten aus zentralen Notaufnahmen und Kindernotaufnahmen aus dem Bundesgebiet die häufigsten Vorstellungsgründe von Kindern und Jugendlichen zu analysieren.

## Methode

Im Rahmen einer retrospektiven deskriptiven Querschnittsstudie wurden aus einem Zeitraum vom 01.01.2019 bis zum 30.06.2022 Behandlungsdaten von Patienten unter 18 Jahren aus 22 Notaufnahmen, darunter 3 Kindernotaufnahmen, ausgewertet. Diese Notaufnahmen verteilen sich auf 8 Bundesländer, von denen 3 in den neuen Bundesländern liegen. Die darunter befindlichen Kindernotaufnahmen befinden sich in Schleswig-Holstein, Niedersachen und Baden-Württemberg. Sieben der Notaufnahmen befinden sich in mittelgroßen Städten, die anderen in Großstädten. Die Nutzung dieser Daten wurde beim Data Use and Access Committee des AKTIN-Notaufnahmeregister [5] beantragt (ID2022_006 und ID2022_007), nach Prüfung der Anfrage wurden die Daten in aggregierter Form zur Verfügung gestellt.

Die Datengewinnung des AKTIN-Notaufnahmeregisters erfolgt durch die in den Notaufnahmen routinemäßig erhobenen Basisdatensätze, die im Krankenhausinformationssystem (KIS) gespeichert und dann in ein lokales Data Warehouse pseudonymisiert exportiert werden. Dort werden die Daten überprüft und in das lokale AKTIN Data Warehouse übertragen.

Ein positives Ethikvotum seitens der Medizinischen Fakultät der Christian-Albrechts-Universität zu Kiel liegt vor (D 449/23).

Insgesamt 251.570 Fälle gingen in die Auswertung ein, davon 223.834 Fälle aus zentralen Notaufnahmen (ZNA) und 27.736 Fälle aus Kindernotaufnahmen (PINA).

Es wurden die 10 häufigsten Vorstellungsgründe nach der Canadian Emergency Department Information System—Presenting Complaint List (CEDIS-PCL) in der deutschen Übersetzung analysiert [6]. Die Vorstellungsgründe wurden detailliert in Bezug zum Alter, Geschlecht, Aufnahmeuhrzeit sowie zum Transport (bodengebunden – Rettungswagen oder Krankentransportwagen; Lufttransport – Rettungstransporthubschrauber) bzw. zu Vorstellungen ohne Transport und der Dringlichkeitseinschätzung nach Manchester Triage System (MTS) und Emergency Severity Index (ESI) ausgewertet.

Im Anschluss wurde die führende ICD-10-Diagnose in der Notaufnahme und die Abschlussdiagnose im Rahmen der Fallpauschalenabrechnung nach § 21 Krankenhausentgeltgesetz (KHEntgG) betrachtet.

Die Altersgruppen wurden wie folgt festgelegt [7]:

- Neugeborenes bis zum vollendeten 28. Lebenstag;
- Säugling ab Beginn des 29. Lebenstages bis zum vollendeten 12. Lebensmonat;
- Kleinkind ab Beginn des 2. bis zum vollendeten 3. Lebensjahr;
- Kinder ab Beginn des 4. bis zum vollendeten 12. Lebensjahr;
- Jugendlicher ab Beginn des 13. bis zum vollendeten 18. Lebensjahr.

Die deskriptiv-statistische Analyse erfolgte unter Angabe der absoluten und relativen Häufigkeiten. Die Datenaufbereitung und statistische Analyse wurde mit der Software „R“ durchgeführt (R Core Team, Foundation for Statistical Computing, Vienna, Austria, 2024; [8]).

## Ergebnisse

### Vorstellungsgründe

**Abb. 1:**
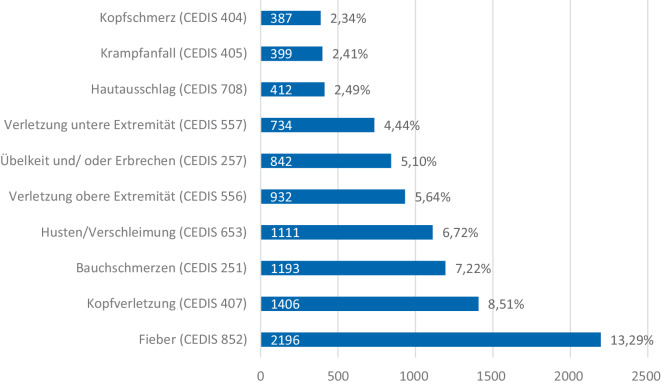
Die 10 häufigsten Vorstellungsgründe nach CEDIS in Kindernotaufnahmen

In Abb. 1 sind die Vorstellungsgründe der 3 Kindernotaufnahmen (PINA) dargestellt. Insgesamt stellen sich hier 58,2 % aller Patienten mit einem der 10 häufigsten Vorstellungsgründe vor.

**Abb. 2:**
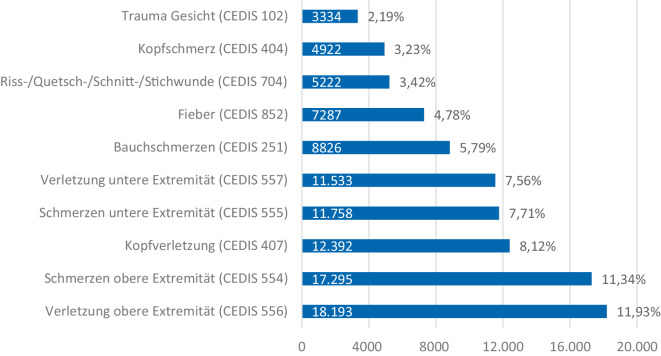
Die 10 häufigsten Vorstellungsgründe nach CEDIS in zentralen Notaufnahmen

Die 10 häufigsten Vorstellungsgründe nach CEDIS-PCL in den zentralen Notaufnahmen (ZNA) sind in Abb. 2 dargestellt. Insgesamt stellen sich hier 66 % der Patienten mit einer dieser 10 Beschwerden in der ZNA vor.

**Abb. 3:**
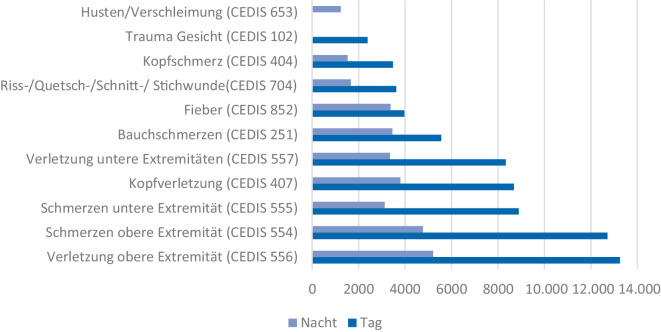
Die häufigsten Vorstellungsgründe in allen Notaufnahmen im Tag-Nacht-Vergleich

In der Kindernotaufnahme finden sich vermehrt nichttraumatologische Gründe, die zu einer Vorstellung führen. Lediglich die 3 Vorstellungsgründe Verletzung der oberen bzw. unteren Extremität sowie Kopfverletzungen lassen auf eine traumatologische Ursache schließen. Anders ist dies in der ZNA. Hier lassen 7 Vorstellungsgründe auf eine traumatologische Ursache schließen und im Gegensatz dazu sind in dieser Kohorte die 3 nichttraumatologischen Gründe Bauchschmerzen, Fieber und Kopfschmerz (Einteilung der CEDIS nach traumatologisch und nichttraumatologisch siehe Supplements Tab. 1).

### Altersgruppenverteilung

Bei der Betrachtung der Altersverteilung bilden die Altersgruppen Kinder (35,2 %) und Säuglinge (27,2 %) zusammen über 50 % der Patientenklientel in Kindernotaufnahmen ab. Im Gegensatz dazu machen in zentralen Notaufnahmen die Altersgruppen Kinder (39,6 %) und Jugendliche (31,6 %) über 70 % der Patienten unter 18 Jahren aus. Neugeborene machen in allen Notaufnahmen den geringsten Teil der Patienten aus (1,2 %; Supplements, Abb. 1). Die Tab. 1 stellt die 3 häufigsten Vorstellungsgründe aufgeteilt nach Altersgruppen und Notaufnahmestruktur dar.

**Tab. 1:** Die 3 häufigsten Vorstellungsgründe nach CEDIS im Vergleich von PINA und ZNA in Abhängigkeit von den Altersgruppen

Alter	Nr.	PINA	ZNA
*Neugeborene*	*1*	Fieber (26,6 %)	Fieber (36,6 %)
	*2*	Husten (25,8 %)	Bauchschmerzen (30 %)
	*3*	Hautausschlag (23,4 %)	Kopfverletzung (10,1 %)/Kopfschmerz (10,1 %)
*Säuglinge*	*1*	Fieber (39 %)	Fieber (28,3 %)
	*2*	Husten (18 %)	Kopfverletzung (20,7 %)
	*3*	Kopfverletzung (17,6 %)	Schmerzen der oberen Extremität (10,3 %)
*Kleinkinder*	*1*	Fieber (27,4 %)	Kopfverletzung (21,8 %)
	*2*	Kopfverletzung (17,4 %)	Verletzung der oberen Extremität (15,5 %)
	*3*	Husten (16,6 %)	Schmerzen der oberen Extremität (15 %)
*Kinder*	*1*	Bauchschmerz (18,8 %)	Schmerzen der oberen Extremität (19,5 %)
	*2*	Verletzung der oberen Extremität (15,5 %)	Verletzung der oberen Extremität (19,4 %)
	*3*	Kopfverletzungen (14 %)	Kopfverletzung (11,9 %)
*Jugendliche*	*1*	Bauchschmerz (26 %)	Verletzung der oberen Extremität (20,6 %)
	*2*	Verletzung der oberen Extremität (16,4 %)	Schmerzen der oberen Extremität (17,6 %)
	*3*	Verletzung der unteren Extremität (14,9 %)	Verletzung der unteren Extremität (16,6 %)

### Geschlecht

In der Geschlechterverteilung überwiegen männliche Patienten mit 55,8 % in den ZNA und 55,3 % in den PINA (Supplements, Abb. 2).

Bei der Betrachtung der Vorstellungsgründe in Abhängigkeit vom Geschlecht fällt auf, dass bei allen Gründen, die auf ein Trauma zurückzuführen sind, das männliche Geschlecht überwiegt. Bei nichttraumatologischen Ursachen ist das weibliche Geschlecht bei 3 der 10 häufigsten Vorstellungsgründe häufiger vertreten: Bauchschmerzen (ZNA weiblich (w)/männlich (m): 56,4 %/43,6 %; PINA (w/m): 53,8 %/46,2 %), Kopfschmerzen (ZNA (w/m): 50,2 %/49,8 %; PINA (w/m): 54,4 %/45,6 %), Krampfanfall (ZNA (w/m): nicht unter den TOP10; PINA (w/m): 52,9 %/47,1 %).

### Transportmittel

Der größte Teil der Patienten (85,5 %) kommt fußläufig in die Notaufnahme, dabei unterscheiden sich zentrale Notaufnahmen (85,3 %) und Kindernotaufnahmen (87,0 %) nicht (Supplements, Abb. 3).

Bezogen auf die 10 häufigsten Vorstellungsgründe ist kein gehäufter Transport mittels Rettungswagen oder Rettungshubschrauber gegenüber fußläufiger Vorstellung zu beobachten. Eine Ausnahme stellt der Krampfanfall dar, bei dem 52,6 % der Fälle via Rettungswagen und 7,7 % der Fälle via Rettungshubschrauber in die Kindernotaufnahme gebracht werden.

Der Anteil von Patienten, die mit einem Rettungshubschrauber in eine Notaufnahme gebracht werden, ist prozentual in einer PINA mit 1,9 % im Vergleich zur ZNA mit 0,2 % höher.

### Notaufnahmediagnose nach ICD 10

In Tab. 2 werden die führenden ICD-Notaufnahmediagnosen benannt, mit denen die Patienten aus der Notaufnahme entlassen werden. Nach Ausschluss fehlender Werte konnten 178.709 Fälle aus 21 Kliniken ausgewertet werden.

**Tab. 2:** Die häufigsten ICD-10-Diagnosen in PINA und ZNA

	ICD-10-Code	Bezeichnung	Fallzahl	Prozent (%)
*PINA* (*n* = 23.807)				
1	S00	Oberflächliche Wunde Kopf	1413	5,9
2	A09	Sonstige Gastroenteritis/Kolitis	1227	5,2
3	J06	Akuter Infekt oberer Atemwege	1016	4,3
4	S01	Offene Wunde Kopf	879	3,7
5	S06	Intrakranielle Verletzung	765	3,2
6	R10	Bauch- Beckenschmerzen	722	3,0
7	J20	Akute Bronchitis	471	2,0
8	R50	Fieber	416	1,8
9	R56	Krämpfe, nicht klassifiziert	410	1,7
10	T14	Verletzungen nicht näher bezeichnet	407	1,7
*ZNA* (*n* = 154.902)				
1	S01	Offene Wunde Kopf	9928	6,4
2	S00	Oberflächliche Wunde Kopf	9094	5,9
3	S06	Intrakranielle Verletzung	5942	3,8
4	S60	Oberflächliche Wunde Hand	5706	3,7
5	S93	Luxation, Zerrung, Verstauchung, Bänder oberes Sprunggelenk	5250	3,4
6	S52	Fraktur Unterarm	5135	3,3
7	A09	Sonstige Gastroenteritis/Kolitis	4597	3,0
8	R10	Bauch‑, Beckenschmerzen	4477	2,9
9	J06	Akuter Infekt oberer Atemwege	4067	2,6
10	T14	Verletzung nicht näher bezeichnet	3442	2,2

Die 10 häufigsten Notaufnahmediagnosen sind auf 32,5 % (PINA) bzw. 37,2 % (ZNA) der Patienten verteilt.

### Zeitliche Vorstellung

**Abb. 4:**
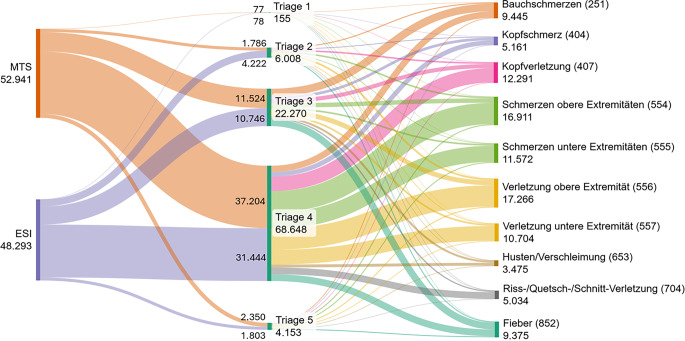
Sankey-Diagramm der 10 häufigsten Vorstellungsgründe in deutschen Notaufnahmen bei Kindern und Jugendlichen nach den 5‑stufigen Triagekategorien MTS und ESI

Für die Analyse der zeitlichen Verteilung der Vorstellungsgründe wurden 173.375 Fälle aus 19 Notaufnahmen, darunter 3 Kindernotaufnahmen, ausgewertet. Die zeitliche Trennung liegt bei 6.00 bis 18.00 Uhr (Tag) und 18.00 bis 6.00 Uhr (Nacht). In der Zeit von 06.00 bis 18.00 Uhr stellten sich 67,0 % der Patienten vor, dagegen 33,0 % in den Abend- und Nachtstunden (Abb. 3) (Supplements, Abb. 4 und 5).

Nichttraumatologische Vorstellungsgründe, insbesondere Fieber (CEDIS 852), nehmen im Verhältnis zu den traumatologischen Vorstellungsgründen in derselben Zeit leicht zu.

### Vorstellungsgründe triagiert nach Manchester Triage und Emergency Severity Index

In Abb. 4 sind in einem Sankey-Diagramm die 10 häufigsten Vorstellungsgründe in deutschen Notaufnahmen, kategorisiert nach Triagestufen des Manchester Triage Systems (MTS) und des Emergency Severity Index (ESI), gezeigt. Insgesamt wurden 52.941 Fälle mit MTS und 48.293 Fälle mit ESI erfasst. Bei der ESI-Triagierung wurden häufiger die Fälle der Triagestufe 2 zugeordnet als bei MTS (MTS *n* = 1786, ESI *n* = 4222). Die Triagekategorie 1 war mit 155 Fällen vertreten und stellte in der Studienkohorte einen Anteil von 0,2 %. Bei der Triagekategorie 2 waren vor allem der CEDIS-Code Bauchschmerzen, die Kopfverletzung und Verletzung der oberen Extremität am häufigsten vertreten. Die meisten Kindernotfälle wurden jedoch in beiden Triagierungssystemen den Triagestufen 3 oder 4 zugeordnet.

## Diskussion

Mit Blick auf die gegenwärtige Krankenhaus- und Notfallreform und zunehmender Belastung von Notaufnahmen [9] ist es von besonderer Relevanz herauszuarbeiten, aus welchen gesundheitlichen Anlässen Kinder und Jugendliche eine Notaufnahme oder Kindernotaufnahme aufsuchen. Nur mittels eines guten Überblicks und genauen Wissens um die Bedürfnisse der Bevölkerung können die knappen Ressourcen des Gesundheitssystems effektiv gesteuert und in eine qualitativ hochwertige Behandlung für Kinder eingesetzt werden. Die hier vorgestellte retrospektive Analyse von Behandlungsdaten aus zentralen und Kindernotaufnahmen des AKTIN-Notaufnahmeregisters gibt einen ersten Überblick über die häufigsten Vorstellungsgründe von Kindern und Jugendlichen.

Betrachtet man die Vorstellungsgründe in den Kindernotaufnahmen im Vergleich zu den in den zentralen Notaufnahmen, lässt sich feststellen, dass in den Kindernotaufnahmen gehäuft Patienten vorgestellt werden, deren Vorstellungsgrund nicht auf eine traumatologische Ursache schließen lässt. Dieser Tatsache können unterschiedliche Ursachen zugrunde liegen. Zum einen ist es denkbar, dass Eltern bei Verletzungen der Kinder weniger die Notwendigkeit einer speziellen pädiatrischen Behandlung sehen, zum anderen unterscheidet sich der Umgang mit pädiatrischen Patienten in den einzelnen Krankenhäusern. Es gibt viele Modelle, die die Aufnahme der Notfallkinder an verschiedenen Orten im Krankenhaus regelt, sodass es dadurch zu einer Verzerrung der Daten zugunsten der traumatologischen Vorstellungsgründen in der Erwachsenennotaufnahme kommt. So gibt es in einigen Krankenhäusern die Regelung, traumatologisch-pädiatrische Patienten in der zentralen Notaufnahme aufzunehmen und zu behandeln, nichttraumatologische Patienten jedoch direkt auf der Kinderstation oder Kindernotaufnahme (wenn vorhanden) aufzunehmen und zu behandeln. Andere Krankenhäuser lassen alle Patienten in der Notaufnahme registrieren. Andere haben eine spezielle Kindernotaufnahme, die traumatologischen Patienten werden jedoch überwiegend in den zentralen Notaufnahmen behandelt, da meist dort die unfallchirurgische Expertise mitverankert ist. Dennoch werden auch in den zentralen Notaufnahmen Kinder und Jugendliche mit nichttraumatologischen Ursachen, wie z. B. Bauchschmerz oder Atemwegsinfekt, vorstellig. Gerade die Entfernung zu Krankenhäusern mit einer Fachabteilung für Kinder und Jugendmedizin, aber auch die wohnortnahe Erstbehandlung in einer ZNA könnten Gründe sein, dass Kinder und Jugendliche dort (erst-)behandelt werden. Der notfallmedizinische Vorstellungsort ist vielfältig und es bedarf einer genaueren Untersuchung, um Optimierungsvorschläge und Qualifikationsanforderungen an das Personal effektiv treffen zu können. In diese Überlegungen sollte auch die Art und Weise, wie die Patienten in die Notaufnahme kommen, noch einmal genauer untersucht werden. Um die Datenlage in der Notfallversorgung für Kinder und Jugendliche zu verbessern, fördert der gemeinsame Bundesauschuss (G-BA) mit „EDCareKids“ ein Innovationsfondprojekt, das zum Ziel hat, die Versorgungsrealität darzustellen [10].

In der Geschlechtsverteilung überwiegt knapp das männliche Geschlecht, was sich durch die um etwa 5 % erhöhte Geburtenrate beim männlichen gegenüber dem weiblichen Geschlecht erklären lässt [11].

Darüber hinaus zeigt die Studie, dass die meisten Patienten selbständig die Notaufnahme aufgesucht haben. In der vorliegenden Auswertung ist der Krampfanfall der einzige Vorstellungsgrund, bei dem die Zuführung der Patienten durch Rettungsdienstfachpersonal die Anzahl der fußläufigen Patienten übersteigt. Nicht erfasst wurde, welche Maßnahmen von den Eltern zuvor getroffen wurden, um eventuell den Besuch in der Notaufnahme zu umgehen (z. B. Vorstellung beim niedergelassenen Kinderarzt oder beim ärztlichen Bereitschaftsdienst). Die Studie von Löber et al. aus einer Metropolregion aus dem Jahr 2019 zeigte bereits, dass alternative Versorgungsstrukturen in der Bevölkerung nur bei etwa einem Drittel bekannt waren und dass diese gehäuft nicht erreichbar waren oder die Patienten direkt an die Notaufnahmen verwiesen wurden [12]. Anhand der führenden Vorstellungsdiagnosen und der Kenntnis über das Unwissen der ambulanten Strukturen in der Bevölkerung sollte auch eine bessere ambulante Versorgung und Erreichbarkeit der ambulanten Strukturen über ein KINZ hinaus überdacht werden. Eine frühzeitige Gesundheitserziehung in den unterschiedlichen Schulstufen könnte hier weiter diese Lücke in Zukunft schließen.

Im Tag-Nacht-Vergleich tritt ein erwartbarer Rückgang der Vorstellungen zur Nacht hinein. Ebenso nehmen die Fälle von Vorstellungsgründen, die auf eine traumatologische Ursache zurückzuführen sind, in den Abend- und Nachtstunden ab, was auf das erhöhte Verletzungsrisiko von Kindern und Jugendlichen aufgrund ihrer Tagesabläufe (Straßenverkehr, Schule/Kita, Sport etc.) am Tag im Vergleich zur Nacht zurückgeführt werden kann.

Bei den 10 häufigsten Vorstellungsgründen und deren Dringlichkeitseinschätzung nach ESI oder MTS stellen dringliche Kindernotfälle mit knapp 6 % in den Triagekategorien 1 und 2 eine kleine Gruppe dar. Ähnlich geringe hochdringliche Fallzahlen konnte die monozentrische OBSERvE-DUS-PED-Studie in einem 3‑Jahres-Zeitraum in ihrer Schockraumversorgung darstellen [13].

Aus diesem Grund bedarf es einer systematischen und multizentrischen Analyse der Versorgung kritisch kranker traumatologischer und nichttraumatologischer Kindernotfälle in Deutschland, jedoch zeigen erste publizierte Überlegungen von Tautz et al. [14] zur Herausforderung der Versorgung gerade dieses pädiatrischen Patientenkollektivs in Bezug zur Ausstattung, der Personalzusammensetzung und Qualifikation in Zentralen Notaufnahmen und Kindernotaufnahmen die Ideen dar [14].

## Limitationen

Die vorliegende Arbeit weist einige Limitationen auf. Zum Zeitpunkt der Abfrage lagen die Daten aus nur 3 Kindernotaufnahmen vor. Damit ist eine umfassende und repräsentative Darstellung per se von Kindernotaufnahmen durch das Register nur eingeschränkt möglich.

Die lokalen Strukturen und Gegebenheiten (z. B. gibt es parallel zur ZNA an den Standorten auch Kindernotaufnahmen) konnten aufgrund der pseudonymisierten Registerauswertung zum Erhebungszeitpunkt nicht abgebildet werden.

Dennoch repräsentiert die vorliegende Analyse die erste multizentrische Arbeit zum Thema Vorstellungsgründe und Tagesrhythmik von Kindernotfällen in deutschen Notaufnahmen.

## Fazit

- Über die Hälfte der Kindernotfallpatienten werden mit einem der 10 häufigsten Vorstellungsgründe in einer Notaufnahme vorgestellt.
- Besonders traumatologische Gründe führen zu einer Vorstellung von Kindern und Jugendlichen in der zentralen Notaufnahme. Jedoch müssen alle Notaufnahmen, auch diejenigen, die nicht auf Kinder und Jugendliche spezialisiert sind, eine Vielzahl von Kindern und Jugendlichen mit nichttraumatologischen Vorstellungsgründen behandeln.
- Kinder und Jugendliche kommen im überwiegenden Teil ohne Rettungsdienst in die Notaufnahme.
- Von den 10 häufigsten Vorstellungsgründen sind knapp 6 % der Kindernotfälle aus den Triagierungskategorien 1 und 2.
- Der Krampfanfall stellt den einzigen der Top-10-Vorstellungsgründe dar, der überwiegend mit dem Rettungsdienst vorgestellt wird.

## Supplementary Information

Supplement

## Data Availability

Die erhobenen Datensätze können auf begründete Anfrage in anonymisierter Form beim korrespondierenden Autor angefordert werden. Die Daten befinden sich im Trusted Data Analytics Center des AKTIN-Notaufnahmeregisters, Institut für medizinische Informatik, Uniklinik RWTH Aachen, Pauwelsstraße 30, 52074 Aachen.
