# Supplementary material for: Children and adolescents in German emergency departments: The most common reasons for presentation throughout the day—current insights from the AKTIN emergency department registry
Source: Med Klin Intensivmed Notfmed. 2025 Mar 6;121(3):193–200. [Article in German] doi: 10.1007/s00063-025-01254-z (PMC13038791; doi:10.1007/s00063-025-01254-z)
Supplement: Supplementary file 1 — Supplement [file 63_2025_1254_MOESM1_ESM.docx]

**Supplement**

Tabelle 1:

| **Eher traumatologisch** | **Eher nicht-tramatologisch** |
| --- | --- |
| 102 Trauma Gesicht | 251 Bauchschmerzen |
| 407 Kopfverletzung | 257 Übelkeit und/oder Erbrechen |
| 554 Schmerzen obere Extremität | 404 Kopfschmerz |
| 555 Schmerzen untere Extremität | 405 Krampfanfall |
| 556 Verletzung obere Extremität | 653 Husten/Verschleimung |
| 557 Verletzung untere Extremität | 708 Hautausschlag |
| 704 Riss-/Quetsch-/Schnitt-/Stichwunde | 852 Fieber |

Tabelle 1: CEDIS Vorstellungsgründe sortiert nach eher auf eine traumatologische Ursache zurück zu führen und eher auf eine nicht-traumatologische Ursache zurückzuführen

Abbildung 1:

Abb. 1: Verteilung des Alters in allen Notaufnahmen, in der PINA und in der ZNA

Abbildung 2:

Abb. 2: Geschlechtsverteilung in allen Notaufnahmen, in der PINA und in der ZNA

Abbildung 3:

Abb. 3: Verteilung der Zuführungswege in alle Notaufnahme, in der PINA und in der ZNA

Abbildung 4:

Abb. 4: Die zeitliche Verteilung der Vorstellungsgründe in Kindernotaufnahmen

Abbildung 5:

Abb. 5: Die zeitliche Verteilung der Vorstellungsgründe in zentralen Notaufnahmen
